# Within-Host Competition Drives Selection for the Capsule Virulence Determinant of *Streptococcus pneumoniae*

**DOI:** 10.1016/j.cub.2010.05.051

**Published:** 2010-07-13

**Authors:** Elena S. Lysenko, Rebeccah S. Lijek, Sam P. Brown, Jeffrey N. Weiser

**Affiliations:** 1 Department of Microbiology, University of Pennsylvania School of Medicine, Philadelphia, PA 19104, USA; 2 Department of Pediatrics, University of Pennsylvania School of Medicine, Philadelphia, PA 19104, USA; 3 Department of Zoology, University of Oxford, South Parks Road, Oxford OX1 3PS, UK

**Keywords:** MICROBIO, EVO_ECOL

## Abstract

For many opportunistic pathogens, it is unclear why their virulence determinants and expression of pathogenic behavior have evolved when damage or death of their host offers no obvious selective advantage to microbial growth or survival [1–3]. Many pathogens initiate interactions with their host on mucosal surfaces and must compete with other members of the microflora for the same niche. Here we explore whether competitive interactions between microbes promote the acquisition of virulence characteristics. During model murine nasal colonization, *Haemophilus influenzae* outcompetes another member of the local flora, *Streptococcus pneumoniae*, by recruiting neutrophils and stimulating the killing of complement-opsonized pneumococci [4]. For *S. pneumoniae*, resistance to opsonophagocytic killing is determined by its polysaccharide capsule [5, 6]. Although there are many capsule types among different *S. pneumoniae* isolates that allow for efficient colonization, virulent pneumococci express capsules that confer resistance to opsonophagocytic clearance. Modeling of interspecies interaction predicts that these more virulent *S. pneumoniae* will prevail during competition with *H. influenzae*, even if production of a capsule is otherwise costly. Experimental colonization studies confirmed the increased survival of the more virulent *S. pneumoniae* type during competition. Our findings demonstrate that competition between microbes during their commensal state may underlie selection for characteristics that allow invasive disease.

## Highlights

► Microbes co-opt host responses to outcompete other species (IM, immunomodulation) ► IM selects for microbial factors that promote resistance to host responses ► Resistance by colonizing *S. pneumoniae* to IM correlates with its capsule type ► Virulence traits like capsule type are selected by IM during mucosal competition

## Results and Discussion

### Theoretical Modeling

One of the most basic and important evolutionary questions posed about pathogens is why they harm the very sources of their livelihoods, their hosts. Theoretical models (reviewed in [7]) frequently assume that traits contributing to virulence (reduced host fitness) provide a net selective advantage to the pathogen within the host (e.g., increasing growth rate) and/or in transmission to another host (e.g., increasing propagule production). However, for many pathogens, these proposed countervailing advantages of virulence are difficult to identify either on the within- or among-host level [1–3]. For example, in the case of the leading pathogen *Streptococcus pneumoniae*, its most common disease states (pneumonia, otitis media, and sepsis) are not contagious conditions and therefore represent a dead end for the organism, especially when the result is the rapid demise of the host [8]. Rather, transmission occurs from the reservoir of pneumococci residing asymptomatically in the nasopharynx during the organism's commensal state [9]. However, among the >92 types of pneumococci expressing structurally distinct capsular polysaccharides, only a few are potentially virulent [10, 11]. So why has the pneumococcus evolved or maintained the capacity for virulent, invasive behavior through the expression of certain thick capsular polysaccharide coats?

To answer this question, we analyzed a simple model for the within-host dynamics of two strains of pneumococcus (resistant *P*_R_ and susceptible *P*_S_) together with *Haemophilus influenzae* (*H*) (Equations 1; see also Supplemental Results available online). In the absence of the resistant strain (Equations 1 with *P*_R_ = 0), we find that the immunomanipulative ability of *H. influenzae* (captured by *x* > 1) increases the within-host market share of *H* up to a point (when *x >* 1/*h*) where *H* can completely outcompete a susceptible lineage of the pneumococcus (Figure S1). The analysis of Equations 1 with *P*_R_ = 0 is consistent with a number of previous theoretical and experimental studies suggesting that immunomanipulation can aid a focal lineage (in our case, *H*) to exclude competitors [4, 12–16] and is more generally consistent with the broad phenomenon of interference competition in bacteria, exemplified by bacteriocins [17].

We now ask, what happens if members of the susceptible lineage (*P*_S_) become resistant to the killing (allelopathic) trait of *H*? “Resistant nonkillers” are widely reported in studies of bacteriocin-mediated interactions [18], and in our focal system some pneumococcal types play the same role, being largely resistant to the immunomanipulation generated by *H. influenzae.* We analyzed the full model (Equations 1) to allow for two distinct lineages of pneumococci, the sensitive lineage *P*_S_, and the resistant lineage *P*_R_. To describe the competitive interactions among the two pneumococcal strains, we introduced the competitive impact parameter *a* and the competitive sensitivity parameter *y* of *P*_R_. When *y* and *a* equal 1, the two pneumococcal strains are competitively equivalent in the absence of *H*. If, however, the acquisition and maintenance of the capsule come at some cost (relative to *P*_S_), then *y >* 1 > *a*, and in the absence of *H*, *P*_S_ will always replace *P*_R_ (*a* < 1 implies an attenuated competitive impact of *P*_R_, and *y* > 1 implies an increased susceptibility to competition in *P*_R_).

**Figure 1:**
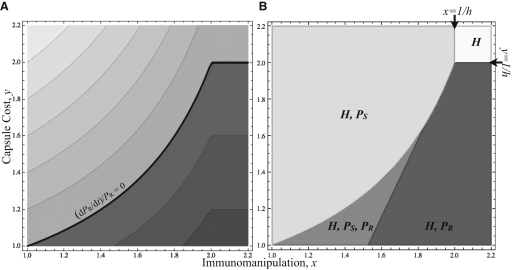
Manipulation by Resident *H. influenzae* Increases Vulnerability to Host Invasion by Resistant Pneumococci *P*_R_ (A) Per capita growth rate of rare *P*_R_ in a host at (*H, P*_S_) equilibrium (Figure S1), as a function of the strength of *H* immunomanipulation (*x*) and the cost of *P*_R_ capsule (*y*). The growth rate (d*P*_R_/dt)/*P*_R_ is positive below the black line; darker shades indicate higher growth rates. (B) The equilibrium (long-term trend) occupants of the nasal mucosa as a function of *x* and *y*. The region supporting nasal establishment by *P*_R_ (dark gray shades) is increasing with *x*. Lightest gray: costly capsule and effective immunomanipulation, only *H* is present. Light gray: costly capsule and weak immunomanipulation, *H* and *P*_S_ are present. Dark gray: cheaper capsule and weak immunomanipulation, all three strains are present. Darkest gray: cheaper capsule and effective immunomanipulation, *H* and *P*_R_ are present. Results are derived from Equations 1 in the Supplemental Results, with parameters *h* = 0.5, p = 0.4, and *a* = 0.6. The exact invasion and equilibrium conditions are detailed in the Supplemental Results.

What happens when we allow the presence of all three lineages? The simplest scenario occurs if *H* is strongly immunomanipulative and is able to entirely exclude *P*_S_ (*x* > 1/*h*; see Figure S1). In this case, the resulting pure *H* equilibrium can be invaded by rare *P*_R_ if *y* < 1/*h* (Figure 1A), leading to a stable coexistence between *H* and *P*_R_ (Figure 1B). The second scenario occurs if *H* is only moderately immunomanipulative, leading first to a coexistence of *H* and *P*_S_ (*x* < 1*/h*; see Figure S1). In this scenario, the resulting (*H*, *P*_S_) equilibrium can then be invaded by rare *P*_R_ if *x* > (y(1+h(1−p))−1)/(h(y−p)) (i.e., *P*_R_ invades more readily as *x* increases; Figure 1A), resulting in either (*H*, *P*_R_, *P*_S_) or (*H*, *P*_R_) coexistence (Figure 1B). Thus, despite a potential competitive disadvantage with *P*_S_ in the absence of *H*, *P*_R_ has an increasing advantage over *P*_S_ as the density and/or immunomanipulative behavior of *H* increases, because it can resist the inflammatory response generated by *H.* In general, selection favors *P*_R_ over *P*_S_ whenever *H*(*x-y*)*h* > *P*_S_(*y* − 1) + *P*_R_(1 − *a*); thus, sufficient *H* can always drive selection toward *P*_R_, given *x > y* (for a more complete analysis, see Supplemental Results). In the limiting case in which *P*_R_ is rare and the cost of capsule is negligible (*y* tends to 1), the more resistant lineage can invade for any amount of immunomanipulation, i.e., whenever *x* > 1.

### Pneumococcal Strains for Experimental Comparison

**Figure 2:**
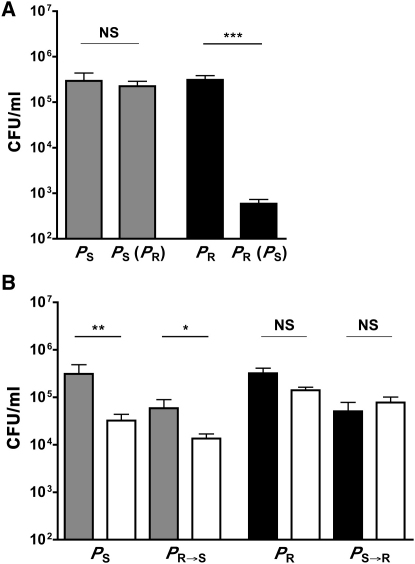
Competition between Pneumococcal Isolates of Different Serotypes and Effect of Capsule Type on Immunomanipulation (A) In the absence of *H. influenzae*, *P*_R_ is competitively inferior to *P*_S_. The density of *S. pneumoniae* (*P*) in upper respiratory tract lavages was determined at 24 hr postintranasal inoculation. Colonization density was compared for *P* strains with type 23F (gray bars, *P*_S_) or type 4 (black bars, *P*_R_) capsules, alone or together (coinoculated *P* strain indicated in parentheses). (B) The density of *S. pneumoniae* (*P*) in the upper respiratory tract was determined at 24 hr postintranasal inoculation, alone without (filled bars) or together with (open bars) *H. influenzae* (*H*). Colonization density was determined for *S. pneumoniae* strains with type 23F (gray bars, *P*_S_ and *P*_R→S_) or type 4 (black bars, *P*_R_ and *P*_S→R_) capsules. (Controls *P*_S_ and *P*_R_ shown in A are also shown here to allow for comparison to groups with *H*.) Values represent the mean ± standard deviation (SD) for 6–17 animals per condition. ^∗^p < 0.05, ^∗∗^p < 0.01, ^∗∗∗^p < 0.001. NS denotes nonsignificant.

We tested the model by determining whether pneumococcal capsule type affects the outcome of immunomanipulation by *H. influenzae* during experimental cocolonization. Pneumococcal isolates of two capsular types were compared. Both type 4 and type 23F pneumococci efficiently colonize the upper airway of mice and induce a similar mild acute inflammatory response characterized by the influx of neutrophils into the nasal spaces following intranasal challenge [19]. Type 4 (isolate T4 representing a *P*_R_ strain), but not type 23F (isolate P1121 representing a *P*_S_ strain), pneumococcus causes invasive infection following intranasal challenge of immunocompetent mice [19, 20]. Additionally, there is a >10^8^-fold difference in the LD_50_ for strains of these types following systemic challenge [21]. However, the greater virulence of *P*_R_ is associated with a cost during colonization, as revealed during intraspecies competition with *P*_S_ (Figure 2A). In contrast, there was no significant effect on *P*_S_ from the competition with *P*_R_. Therefore, we determined whether the colonization deficit in *P*_R_ would be offset by its potential advantage in the context of cocolonization by *H. influenzae*, as predicted in the theoretical model.

To address this question, we generated isogenic strains in which the capsule type was switched (type 4 switched to type 23F to generate *P*_R→S_ and type 23F switched to type 4 to generate *P*_S→R_) to control for the potentially confounding effect of genetic background. A sensitive capture ELISA was used to confirm that *P*_R→S_ and *P*_S→R_ produce equivalent levels of capsular polysaccharide to the isotypic strain (data provided in Table S1). When tested for the ability to cause bacteremic infection, the competitive index (CI) for *P*_S→R_/*P*_S_ was >6.0, confirming the contribution of capsule type rather genetic background to virulence.

### The Effect of Capsule Type on Pneumococcal Survival during Experimental Competition

The density of colonizing *H. influenzae* (*H*) is not affected by the presence of *S. pneumoniae*. However, cocolonization of *H* with *P*_S_ results in a steep decline in density of *P*_S_ in the upper airways within 24 hr postinoculation (Figure 2B). In contrast, although *P*_R_ alone colonized at similar levels to *P*_S_ alone in the absence of the competitor, coinoculation with *H* had no significant effect on its colonization. Switching of the capsule type from *P*_S→R_ was sufficient to eliminate its susceptibility to competition, whereas switching the capsule type from *P*_R→S_ was sufficient to render it sensitive to competition by *H*. These results confirmed that capsule type alone is sufficient to dictate the outcome of a competitive interaction with another species.

### Loss of Pneumococci during Competition Requires Opsonophagocytosis

**Figure 3:**
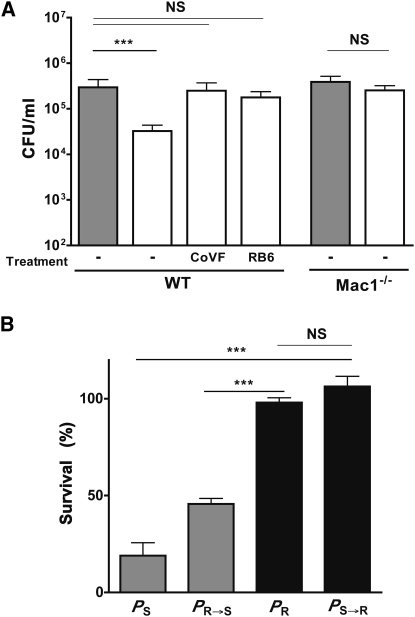
The Immunomanipulative Effect of *H. influenzae* on *S. pneumoniae* Requires Opsonophagocytosis and Is Dependent on Capsule Type (A) The density of *P*_S_ in the upper respiratory tract was determined at 24 hr postintranasal inoculation with the *P*_S_ alone (gray bars) or together with (open bars) *H. influenzae* (*H*). Colonization density was compared in C57BL/6 mice (WT) treated with RB6-8C5 (intraperitoneal [i.p.] antibody treatment to deplete neutrophil-like cells), in cobra venom factor (i.p. cobra venom factor pretreatment to deplete complement), and in Mac1^−/−^ (CD11b/CD18) mice. Values represent the mean ± SD for 4–11 animals per condition. (B) The relationship of capsule type to survival in opsonophagocytic killing assays. Neutrophil-enriched peritoneal exudate cells (PECs) were obtained from mice pretreated by i.p. administration of casein. Survival of the *S. pneumoniae* strains with type 23F (gray bars, *P*_S_ and *P*_R→S_) or type 4 (black bars, *P*_R_ and *P*_S→R_) capsules is shown relative to controls without neutrophil-enriched PECs. Values represent ≥3 independent determinations in duplicate ± SD. ^∗^p < 0.05, ^∗∗^p < 0.01, ^∗∗∗^p < 0.001. NS denotes nonsignificant.

Next, we explored the host mechanisms selecting for capsule type during interspecies competition. In C57BL/6 mice, systemic depletion of neutrophils is sufficient to eliminate the competitive effect of *H. influenzae* on type 23F pneumococci (*P*_S_) (Figure 3A) [4]. Additionally, this competitive effect was no longer observed in mice in which complement activity was depleted by prior treatment with cobra venom factor (CoVF). There was also no competitive effect in mice lacking the complement C3 receptor Mac-1 (CD11b/CD18). Together, the requirement for neutrophils, complement, and the Mac-1 provided evidence that the loss of *P*_S_ during cocolonization was dependent on opsonophagocytic clearance.

To test whether capsule type could account for differences in opsonophagocytic clearance, we compared each of the strains in an ex vivo killing assay using elicited murine neutrophils and serum as a source of complement (Figure 3B). Killing in this assay was dependent on the capsule type rather than genetic background. Strains expressing the avirulent type 23F capsule type (*P*_S_ and *P*_R→S_) were sensitive to the effects of neutrophils and complement. In contrast, no killing was detected in strains expressing the virulent type 4 capsule type (*P*_R_ and *P*_S→R_). Thus, the outcome of interspecies competition requires opsonophagocytosis and correlates with relative resistance to killing by this mechanism. Because capsule type impacts sensitivity to complement deposition and subsequent phagocytosis, it may have a profound effect on the outcome of mucosal competition and, consequently, the selection for more resistant or virulent types [6].

### The Effect of *H. influenzae* on Capsule-Dependent Survival during Experimental Competition

**Figure 4:**
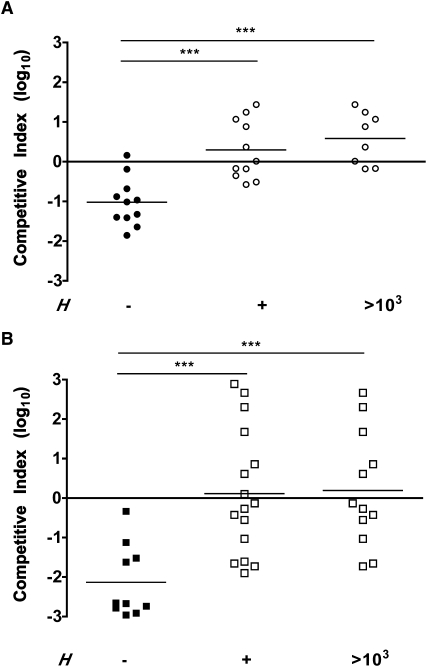
When *S. pneumoniae* with Different Capsules Compete, Immunomodulation by *H. influenzae* Enhances Colonization by the More-Virulent Strain and Inhibits Colonization for the Less-Virulent Strain (A and B) Mice were challenged with equal inocula of 2 isogenic *S. pneumoniae* strains with type 23F (*P*_S_) and type 4 (*P*_S→R_) capsules (A) or 2 *S. pneumoniae* clinical isolates with type 23F (*P*_S_) and type 4 (*P*_R_) capsules (B), and the density of each strain was determined in upper respiratory tract lavages at 24 hr postinoculation. Each symbol represents the competitive index value for an individual animal without (solid symbols) or with (open symbols) *H. influenzae*. The competitive index was calculated based on the ratio of each strain in nasal lavages compared to the ratio in the inoculum. The horizontal line is at a value of 1 (log_10_ = 0). (A) A value less than 1 indicates that *P*_S_ outcompetes *P*_S→R_, and a value greater than 1 indicates that *P*_S→R_ outcompetes *P*_S_. (B) A value less than 1 indicates that *P*_S_ outcompetes *P*_R_, and a value greater than 1 indicates that *P*_R_ outcompetes *P*_S_. Competitive index values were also stratified for animals with high (>10^3^ cfu/ml) density of colonization by *H*. Mean values for each condition are indicated by horizontal bars. ^∗∗∗^p < 0.001. NS denotes nonsignificant.

Next, we tested our model by directly competing the isogenic virulent (*P*_S→R_) and avirulent (*P*_S_) pneumococcal strains in the presence of the immunomodulator, *H. influenzae* (*H*) (three-strain experiment, Figure 4A). In comparison to controls without *H*, colonization density was significantly enhanced for *P*_S→R_ and inhibited for *P*_S_ (p < 0.05 and p < 0.01, respectively). The mean competitive index (strain ratio after in vivo competition, normalized by ratio in inoculum) for *P*_S→R_ /*P*_S_ was significantly <1 without *H* (p < 0.001, t test compared to null expectation of CI = 1). To test the model prediction that increasing densities of *H* will change the magnitude of selection on capsule type, we stratified mice by their colonization density with *H. influenzae*. For mice colonized by *H. influenzae* at a higher (>10^3^ cfu/ml) density, the mean competitive index for *P*_S→R_ /*P*_S_ was significantly >1 (p < 0.05, t test compared to null expectation of CI = 1), supporting our principal prediction that sufficient densities of *H* can generate positive selection for capsule virulence traits in the pneumococcus. Competition between nonisogenic, clinical isolates *P*_S_ and *P*_R_ was more variable, although a significant increase in the competitive index (*P*_R_ /*P*_S_) was also observed during immunomodulation by *H. influenzae* (Figure 4B, p < 0.001). These experimental results demonstrate that immunomodulation by *H. influenzae* can dictate the relative capsule-type-dependent fitness of pneumococcal strains.

### Within-Host Ecology and the Evolution of Virulence

The impact of within-host competition on the evolution of virulence has been the subject of a diverse range of models, offering contrasting explanations for either an increase or a decrease in virulence as within-host diversity increases [22]. A key distinction separating our work from nearly all theoretical studies of virulence evolution in multiple infected hosts is that we examine multispecies infections (as opposed to multiple strains differing only in a focal virulence trait [22]). A major consequence of this distinction is that the direction of selection on virulence traits no longer simply depends on the number of coinfecting strains but now depends very much on their identity. In the context of our current study, we show that more invasive—and therefore more virulent—pneumococcal capsule variants can be selected for within the mucosal environment, but only if there is sufficient burden of an immunomanipulative coinfecting microbe (Figure 4A).

Because different capsule types are antigenically distinct and the immunodominant constituent of the pneumococcal surface, it has been assumed that the diversity of capsular types arose to escape immune pressure in the host [23]. However, only a few capsule types generally account for the overwhelming majority of both carriage and disease isolates, for unknown reasons [11]. There is a metabolic cost to produce large amounts of capsular polysaccharide, and the capsule may obscure surface molecules, such as adhesins, needed to interact with the host [24–26]. We suggest that the diversity of capsule types will in part be explicable by variation among the flora. Thus, in situations in which the competitor (*H*) is highly prevalent and/or highly immunomanipulative (high *H* and/or high *x*), we anticipate that selection would favor thicker capsule types that are more resistant to clearance by opsonophagocytosis. Thus, our observations provide an explanation for the evolution of potentially costly capsule types more resistant to acute inflammatory responses that promote the virulent behavior of an organism with an otherwise commensal lifestyle.

Our findings provide a theoretical and experimental demonstration that the capsule virulence determinant confers a selective advantage during colonization by allowing for persistence during mucosal inflammation induced by competitive interactions among the flora. This paradigm offers a possible explanation for the disproportionate prevalence of the few pneumococcal types associated with invasive disease [27]. Like the pneumococcus, many opportunist pathogens must become established at host sites in the presence of other microbes. We propose that events during within-host competition may underlie the benefit of virulence determinants that either induce or protect against the host's inflammatory responses.

## Experimental Procedures

### Theoretical Model

The results in Figure 1 are derived from the following differential equations, tracking the densities of *H. influenzae* (*H*), and two strains of *S. pneumoniae* (sensitive [*P*_S_] and resistant [*P*_R_]):

$$dH/dt=H\left(1-H-p\;P_{\text{S}}-p\;a\;P_{\text{R}}\right)$$

$$dP_{\text{S}}/dt=P_{\text{S}}\left(1-h\;x\;H-P_{\text{S}}-a\;P_{\text{R}}\right)$$

(Equations 1). $$dP_{\text{R}}/dt=P_{\text{R}}\left(1-h\;y\;H-y\;P_{\text{S}}-P_{\text{R}}\right)$$

For detailed presentation and analysis, see Supplemental Results. The parameters *p*, *a*, *h*, *x*, and *y* capture the competitive interactions among the three strains. *p* is the competitive impact of *P*_S_ on *H*, and *h* is the impact of *H* on *P*_S_ in the absence of immunomanipulation (p = *h* = 1 would imply competitive equivalence; we assume *p* < 1, *h* < 1, ensuring coexistence between *H* and *P*_S_ when *x =* 1). *x* captures the additional impact of *H* on *P*_S_ because of immunomanipulation (*x =* 1 would imply no immunomanipulation; we assume *x* > 1). *y* and *a* capture the competitive abilities of *P*_R_ relative to *P*_S_. We assume *y* > 1 (*P*_R_ is more sensitive to competition from the other strains) and *a* < 1 (*P*_R_ has an attenuated competitive impact on the other strains).

### Mouse Strains and Model of Nasal Colonization

Six- to eight-week-old mice used in the study were housed in accordance with Institutional Animal Care and Use Committee protocols. Mouse strains used for this study included C57BL/6 (WT) and B6.129S4-*Itgam^tm1Myd^*/J (Mac-1 [CD11b/CD18]-deficient mice, Jackson Laboratories), with a targeted mutation in the gene for integrin alpha M or complement receptor type 3 [28]. Neutrophils from these animals are deficient at phagocytosing complement-opsonized particles and in several Fc-mediated functions.

Briefly, groups of at least 5 mice per condition were inoculated intranasally with 1 × 10^7^ cfu of phosphate-buffered saline (PBS)-washed, midlog phase *H. influenzae* or *S. pneumonia*, each applied separately to each naris in 10 μl. Pneumococcal strains and characteristics are provided in the Supplemental Experimental Procedures. Twenty-four hours postinoculation, the animal was sacrificed, the trachea was cannulated, and 200 μl of PBS was instilled. Lavage fluid was collected from the nares for determination of viable counts of bacteria in serial dilutions plated on selective medium, containing antibiotics to inhibit the growth of contaminants. To select for *H. influenzae* (H636), we used streptomycin at a concentration of 100 μg/ml. Neomycin, 20 μg/ml, or erythromycin, 1 μg/ml, was used to select for *S. pneumonia* either P1121 and its derivatives or TIGR4 and its derivatives, respectively.

### Statistical Analysis

For single-factor analyses, statistical comparisons of colonization between groups were made using either the Kruskal-Wallis test with Dunn's post test or t tests, as appropriate.

For statistical comparisons of two or more variables simultaneously, two-way analysis of variance (ANOVA) was used to determine whether the interaction between these variables was significant. If the interaction between variables was significant by two-way ANOVA, Bonferroni post tests were used to determine each variable's effect on colonization outcome. If there was no statistically significant interaction between variables, colonization outcomes were compared using one-factor analyses, as described above. For comparisons of competitive indices (calculated as the ratio of strains in nasal lavages compared to the ratio of strains in the inoculum), log-transformed values were analyzed using ratio t tests.

## Supplemental Information

Document S1. Supplemental Data, Supplemental Experimental Procedures, One Figure, and One Table
