# Supplementary material for: Within-Host Competition Drives Selection for the Capsule Virulence Determinant of Streptococcus pneumoniae
Source: Curr Biol. 2010 Jul 13;20(13):1222–6. doi: 10.1016/j.cub.2010.05.051 (PMC2913241; doi:10.1016/j.cub.2010.05.051)
Supplement: Document S1. Supplemental Data, Supplemental Experimental Procedures, One Figure, and One Table [file mmc1.pdf]

Current Biology, Volume *20*

Supplemental Information

Within-Host Competition Drives Selection for the Capsule Virulence Determinant of *Streptococcus pneumoniae*

Elena S. Lysenko, Rebeccah S. Lijek, Sam P. Brown, and Jeffrey N. Weiser

Supplemental Results

Detailed Presentation of Model and Results

We began by constructing a simple model of the within-host dynamics of *H. influenzae*, competing with various strains of pneumococcus. Our models are based on classic Lotka-Volterra competition equations[1], with maximal growth rates and carrying capacities normalized to 1 (ie, a rare single-strain inoculate would initially grow exponentially and then more slowly until net growth would stop at carrying capacity, which here is the unit of density, ie 1). Specifically, let the variables H and P represent the within-host densities of the two species (scaled to their respective carrying capacities), then their change in time can be represented by the following differential equations

$$dH/dt = H(1 - H - p\,P)$$
$$dP/dt = P(1 - h\,x\,H - P) \qquad \text{[Equations S1]}$$

The competitive interactions among the two strains are captured by the coefficients, p, h and x. These parameters capture the relative competitive force that one strain imposes on the other. If $p = h = x = 1$, then the species are competitively equivalent, one *H. influenzae* is as much of a competitive burden as one pneumococcus, from the perspective of either species. Here we assume that the two species are incompletely competitively differentiated, using somewhat different resources and/or micro-environments, therefore tending to favor coexistence. Specifically we assume that the baseline competition parameters p and h are less than one (p is the relative competitive pressure of pneumococci on *H. influenzae*, and h is the reverse effect), so that in the absence of any immune-mediated competitive effects (i.e. when $x = 1$) we will have coexistence (each will have an advantage when rare, due to distinct resource use, Fig. S1).

To account for additional immunomediated competitive impacts of H on P, we allow the immunomanipulation parameter x to increase above 1. Increasing x above 1 can be viewed as representing the negative effect of immunomanipulation on the pneumococcus, triggered by *H. influenzae* [2]. A stability analysis of model 1 illustrates that greater values of x (e.g. due to more

competent immune systems) will tend to increase the equilibrium market share of H, up to a point (when $x > 1/h$) where H can completely outcompete the pneumococcus (Fig. S1).

We now recover the full model presented in the main text (equations 1) by allowing for two distinct lineages of pneumococci, the susceptible lineage P_S and the resistant lineage P_R

$$dH/dt = H(1 - H - p\,P_S - p\,a\,P_R)$$
$$d\,P_S/dt = P_S\,(1 - h\,x\,H - P_S - a\,P_R)\;.$$
$$dP_R/dt = P_R\,(1 - h\,y\,H - y\,P_S - P_R) \qquad \text{[Equations S2]}$$

When $P_R = 0$, we recover model S1, with P_S equivalent to P. To describe the competitive interactions of the new strain P_R, we introduce the competitive impact parameter a, and the competitive sensitivity parameter y of P_R. When y and a equal one, the two pneumococcal strains are competitively equivalent in the absence of H. If however the acquisition and maintenance of the capsule comes at some cost (relative to P_S), then $y > 1 > a$, and in the absence of H, P_S will always replace P_R ($a < 1$ implies an attenuated competitive impact of P_R, and $y > 1$ implies an increased susceptibility to competition in P_R). The principal results of model S2 are presented in the main text, and their derivation is detailed below.

Stability Analysis of the Mathematical Models

Stability analyses were performed following standard analyses of Jacobian matrices[1]. Throughout, the following assumptions are made, as detailed in the main text: $h < 1$, $p < 1$, $y > 1$, $x > 1$, $a < 1$.

***H. influenzae* and Sensitive Pneumococcus Only (Equations S1, Figure S1)**

The first model (equation S1) converges to one of two potentially stable states, dependent on the magnitude of the immuno-manipulative effect of H; either H alone ($H^* = 1$, $P^* = 0$; if $x>1/h$), or coexistence ($H^* = \frac{1-p}{1-hpx}$, $P^* = \frac{1-hx}{1-hpx}$; if $x < 1/h$). The co-existence equilibrium (rephrased as proportion $H = H^*/(H^*+P^*) = \frac{1-p}{2-p-hx}$) is illustrated in figure S1, as a function of p, h and x. When $hx > 1$ (Figure S1), the H alone equilibrium is reached.

Analysis of the Full Model (Equations S2, Figure 1)

We now turn to analyse the full model (also presented as equations 1 in the main text). Selection for resistant capsule will be positive whenever the per-capita growth rate of P_R exceeds that of P_S, ie whenever $(dP_R/dt)/P_R > (dP_S/dt)/P_S$. Using equations S2, we can re-write this inequality as $H(x\text{-}y)h > P_S(y\text{-}1) + P_R(1\text{-}a)$. Note that the selection differential $(dP_R/dt)/P_R - (dP_S/dt)/P_S$ is strictly a positive function of immuno-manipulation x (given $H > 0$) and of *H. influenzae* burden H (given $x > y$). To derive the results in Fig 1A, we now focus on the invasion conditions for rare P_R in a host at a candidate equilibrium defined by the analysis of model 1. Specifically, we look at the per-capita growth rate $(dP_R/dt)/P_R = 1 - hyH^* - yP_s{}^*$, given $\{H^* = \frac{1-p}{1-hpx}$, $P_S^* =$

$\frac{1-hx}{1-hpx}$ if $x < 1/h$, otherwise $H^* = 1$, $P_S^* = 0$}. The resulting expression $\{(dP_R/dt)/P_R = \frac{1-y+h(p(y-x)+y(x-1))}{1-hpx}$ if $x < 1/h$, otherwise $(dP_R/dt)/P_R = 1-hy\}$ is plotted as a function of x and y in Figure 1A.

Understanding the long-term behaviour of the full model is more complex, as it has 7 non-zero equilibria. Given $p < 1$, H can always invade any combination of P_S and P_R strains, so we focus here on the 4 equilibria with H present (figure 1B). The H alone equilibrium ($H^* = 1$, $P_S^* = 0$, $P_R^* = 0$) is stable if $x > 1/h$ and $y > 1/h$. The H and P_R equilibrium ($H^* = \frac{1-ap}{1-ahpy}$, $P_S^* = 0$, $P_R^* = \frac{1-hy}{1-ahpy}$) is stable if $x > \frac{1-a(1-hy(1-p))}{h(1-ap)}$ and $y < 1/h$. The H and P_S equilibrium ($H^* = \frac{1-p}{1-hpx}$, $P_S^* = \frac{1-hx}{1-hpx}$, $P_R^* = 0$) is stable if $x < 1/h$ and $y > \frac{1-hpx}{1-h(p+x-1)}$. Finally, the all-present coexistence equilibrium ($H^* = \frac{1-p}{1-hpx}$, $P_S^* = \frac{1-hx+a(h(px+y-py)-1)}{(1-hpx)(1-ay)}$, $P_R^* = \frac{1-y+h(y(x-1)+p(y-x))}{(1-hpx)(1-ay)}$) is stable if $y < \frac{1-hpx}{1-h(p+x-1)}$ and $x < \frac{1-a(1-hy(1-p))}{h(1-ap)}$.

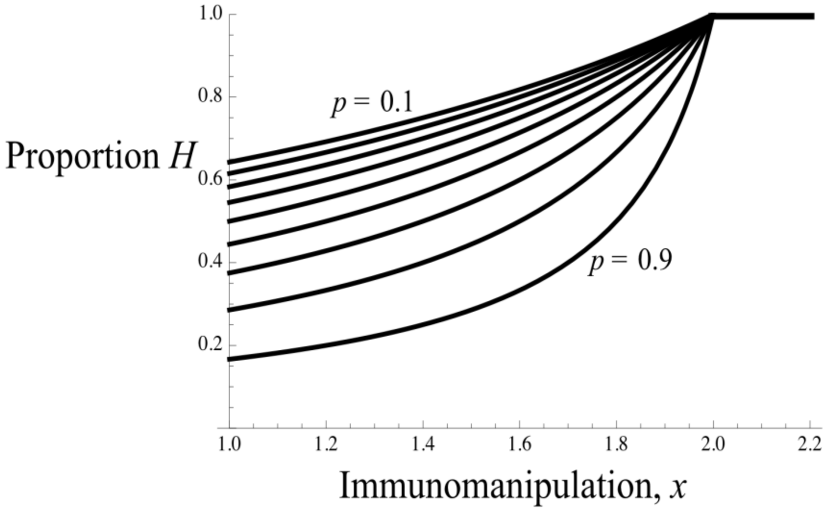

Figure S1. Manipulation (*x*) Increases the Market Share of *H* at the Expense of *P*
The proportion of *H* at equilibrium ($H^*/(H^*+P^*)$, where $\{H^*,P^*\}$ is the coexistence equilibrium of model 1) is plotted as a function of immunomanipulation *x*, for a range of pneumococcal competitiveness (*p* from 0.1 to 0.9) and $h = 0.5$. When $h\,x > 1$, *H* excludes *P*, for any $p < 1$.

Table S1. Pneumococcal Strains and Characteristics

**Strain**	**Capsule Type**	**Genetic Background**	**Capsule Polysaccharide** (ng/10^5 CFU $\pm$ S.D.)	**Reference**
P_S	23F	P1121	0.54 $\pm$ 0.25	[3]
P_R	4	TIGR4	2.50 $\pm$ 0.05	[4]
$P_{S\rightarrow R}$	4	P1121	2.46 $\pm$ 0.14	This study
$P_{R\rightarrow S}$	23F	TIGR4	0.52 $\pm$ 0.18	This study

Supplemental Experimental Procedures

Bacterial Strains and Culture Conditions

H. influenzae and *S. pneumoniae* strains were grown as previously described[5]. *H. influenzae* strain H636 - a type b capsule-expressing, spontaneously streptomycin-resistant mutant of strain Eagan used for studies was selected because of its ability to colonize the murine mucosa[2]. *S. pneumoniae* strains P1121 (a type 23F capsule expressing isolate from the human nasopharynx) and T4 (a type 4 capsule expressing isolate) were chosen for co-infection studies as serotypes either known for prolonged asymptomatic carriage (23F) or well-colonizing but virulent serotype (T4)[3, 4, 6].

An unmarked capsule switch mutant in the T4 (type 4) background created to express the type 23F capsule was generated from an unencapsulated strain using positive selection with Janus cassette technology[7] Strain P1690 (T4 background with 23F capsule locus) was created by transformation of DNA from P1121 into recipient strain P1412 (T4 unencapsulated mutant due to interruption of the locus with insertion of Janus cassette) [6]. The capsule type switch was verified by quelling and prior to testing strain P1690 was mouse passaged by intranasal infection.

Because of its lower transformability, we were unable to utilize this technology in the P1121 strain background. P2140 (23F background with type 4 capsule locus) was prepared in several steps. During the first step, an unencapsulated 23F strain was obtained by transformation with DNA from the T4 strain lacking capsule expression due to the insertion of Janus cassette in the *cps* locus (P1412). This strain (P2109) was back transformed twice with selection for resistance to kanamycin and the lack of capsule was confirmed by PCR and quelling. For the second step random inserts of a spectinomycin resistance cassette into 2.9 kb PCR fragment of T4 capsule loci were prepared using MarC9 transposase[8]. PCR fragment for the transposon mutagenesis was generated using T4 chromosomal DNA and primers cpsF6 (AATCAGGATTTGCAGGCAGGA) and cpsR6 (TTCCGTACCATCTCCAACAAAATG). Third step included transformation of created fragments back into T4 selecting for spectinomycin resistance. Encapsulation of the resulting construct (P2110) was verified by colony morphology and quelling. Finally, 23F strain expressing the type 4 capsule was obtained by transforming P2109 with P2110 chromosomal DNA followed by serial back transformation. Capsule type was confirmed by quelling and 23F background was verified by PCR analysis of *pspA* gene sequence. The created mutant of P1121, P 2140 (type 4 capsule, Km^S, $Spec^R$), was mouse passaged by intranasal infection twice prior to *in vivo* co-colonization studies.

Pneumococcal strains and characteristics are described in Supplemental Information.

The relative virulence of strains was assessed following ip inoculation with a mixed inoculum (10^5-10^7 CFU/animal). Capsule type was determined in blood cultures at 24 hrs post-inoculation followed by immunoblotting. The competitive index was calculated based on the ratio of types in blood compared to the ratio of types in the inoculum.

Isolation and Characterization of Murine Neutrophils

Neutrophil-enriched PECs were isolated as previously described[2]. Briefly, phagocytes were obtained by lavage of the peritoneal cavity (8 ml/animal with Hanks' buffer minus Ca2+ and Mg2+ (Invitrogen) plus 50mM HEPES) of mice treated 24 hr and again 2 hrs prior to cell harvest by i.p. administration of 10% casein in PBS (1 ml/dose). Administration of casein provided for a higher and more consistent yield of cells. Cells collected from the peritoneal cavity lavage (PECs) were enriched for neutrophils using separation by a Ficoll density gradient centrifugation according to the manufacturer's protocol (MP Biomedicals, Irvine, California). Neutrophil-enriched fractions were collected and washed with 5 ml of Hank's buffer without Ca^{++} or Mg^{++} (Gibco, San Diego, California) plus 0.1% gelatin. An aliquot of these cells was characterized using FACS for staining of granulocytes with anti-mouse Gr-1 mAb to Ly6.G (BD Biosciences, San Jose, California) and showed >90% positively-stained cells following enrichment. Additional characterization involved staining for CD11b/CD18 (BD Biosciences, San Jose, California).

Phagocytic Killing Assays

Neutrophil-enriched PECs were counted by trypan blue staining and adjusted to a density of 7 $x10^6$ cells/ml. Killing during a 45 min incubation at 37°C with rotation was assessed by combining 10^2 PBS washed, mid-log phase bacteria (in 10 µl) with complement source (in 20 µl), 10^5 mouse phagocytes (in 40 µl) and Hank's buffer with Ca^{++} and Mg^{++} (Gibco, San Diego, California) plus 0.1% gelatin (130 µl). Complement source consisted of fresh mouse serum from C57Bl/6 mice. After stopping the reaction by incubation at 4°C, viable counts were determined in serial dilutions. The percent killing was calculated by comparison to controls with inactivated complement (56^0C for 30 min) where there was no loss of bacterial viability.

Capture ELISA for Quantifying Capsular Polysaccharide

S. pneumoniae strains with original and switched capsule types were grown aerobically on blood agar plates (BBL) overnight at 37°C in 5% CO_2. Bacteria were suspended in PBS to OD_{620}=0.5, pelleted, re-suspended in 1/10 of the original volume in PBS and sonicated. Polystirene 96-well Immulon 2HB plates (Thermo Electron Co, Milford, MA) were pre-coated with a 1:5000 dilution of rabbit polyclonal typing sera (Statens Serum Institut)) for 4h at 4°C. Bacterial sonicates were added to the plates diluted sequentially in 0.05M carbonate buffer. Purified type 23F and 4 capsular polysaccharides (purchased from ATCC) were used to generate a standard curve. Plates were incubated for 2 hrs at room temperature and followed by a 1 hr incubation with a mAb against type 4 or 23F capsule (provided by Dr. M. Nahm) at a dilution of 1:400. Plates were developed following incubation with goat anti-mouse IgG1 conjugated with alkaline phosphatase (Merck, St. Louis, MO) at a dilution of 1:10,000 for 1 hr at room temperature. Amount of capsular polysaccharide was determined by comparison of optical density of bacterial lysates and polysaccharide standards. Results of capsular polysaccharide quantification assays is provided in Supplemental Table 1.

Supplemental References

1. Otto, S., and Day, T. (2007). A biologist's guide to mathematical modeling in ecology and evolution, (Princeton, NJ: Princeton University Press).
2. Lysenko, E.S., Ratner, A.J., Nelson, A.L., and Weiser, J.N. (2005). The role of innate immune responses in the outcome of interspecies competition for colonization of mucosal surfaces. PLoS Pathogens *1*, 1-9.
3. McCool, T., and Weiser, J. (2004). Limited role of antibody in clearance of *Streptococcus pneumoniae* in a murine model of colonization. Infect Immun *72*, 5807-5813.
4. Tettelin, H., Nelson, K.E., Paulsen, I.T., Eisen, J.A., Read, T.D., Peterson, S., Heidelberg, J., DeBoy, R.T., Haft, D.H., Dodson, R.J., et al. (2001). Complete genome sequence of a virulent isolate of Streptococcus pneumoniae. Science *293*, 498-506.
5. Gould, J.M., and Weiser, J.N. (2002). The inhibitory effect of C-reactive protein on bacterial phosphorylcholine-platelet activating factor receptor mediated adherence is blocked by surfactant. J Infect Dis *186*, 361-371.
6. Nelson, A., Roche, A., Gould, J., Chim, K., Ratner, A., and Weiser, J. (2007). Capsule enhances pneumococcal colonization by limiting mucus-mediated clearance. Infect Immun *75*, 83-90.
7. Sung, C., Li, H., Claverys, J., and Morrison, D. (2001). An rpsL cassette, janus, for gene replacement through negative selection in *Streptococcus pneumoniae*. Appl Environ Microbiol *67*, 5190-5196.
8. Hava, D., and Camilli, A. (2002). Large-scale identification of serotype 4 *Streptococcus pneumoniae* virulence factors. Mol Microbiol *45*, 1389-1406.
